# Automated left atrial strain analysis for predicting atrial fibrillation in severe COVID-19 pneumonia: a prospective study

**DOI:** 10.1186/s13613-021-00955-w

**Published:** 2021-12-07

**Authors:** Christophe Beyls, Alexis Hermida, Yohann Bohbot, Nicolas Martin, Christophe Viart, Solenne Boisgard, Camille Daumin, Pierre Huette, Hervé Dupont, Osama Abou-Arab, Yazine Mahjoub

**Affiliations:** 1 Department of Anesthesiology and Critical Care Medicine, Amiens University Hospital, 1, Rond-point du Pr Cabrol, 80054 Amiens, Cedex 1, France; 2 Department of Rythmology, Amiens University Hospital, 80054 Amiens, France; 3 Department of Cardiology, Amiens University Hospital, 80054 Amiens, France; 4 UR UPJV 7518 SSPC (Simplification of Care of Complex Surgical Patients) Research Unit, University of Picardie Jules Verne, Amiens, France

**Keywords:** Left atrial strain, Atrial fibrillation, COVID-19, Pneumonia, Intensive care unit

## Abstract

**Background:**

Atrial fibrillation (AF) is the most documented arrhythmia in COVID-19 pneumonia. Left atrial (LA) strain (LAS) analysis, a marker of LA contractility, have been associated with the development of AF in several clinical situations. We aimed to assess the diagnostic ability of LA strain parameters to predict AF in patients with severe hypoxemic COVID-19 pneumonia. We conducted a prospective single center study in Amiens University Hospital intensive care unit (ICU) (France). Adult patients with severe or critical COVID-19 pneumonia according to the World Health Organization definition and in sinus rhythm were included. Transthoracic echocardiography was performed within 48 h of ICU admission. LA strain analysis was performed by an automated software. The following LA strain parameters were recorded: LA strain during reservoir phase (LASr), LA strain during conduit phase (LAScd) and LA strain during contraction phase (LASct). The primary endpoint was the occurrence of AF during ICU stay.

**Results:**

From March 2020 to February of 2021, 79 patients were included. Sixteen patients (20%) developed AF in ICU. Patients of the AF group were significantly older with a higher SAPS II score than those without AF. LAScd and LASr were significantly more impaired in the AF group compared to the other group (− 8.1 [− 6.3; − 10.9] vs. − 17.2 [− 5.0; − 10.2] %; *P* < 0.001 and 20.2 [12.3;27.3] % vs. 30.5 [23.8;36.2] %; *P* = 0.002, respectively), while LASct did not significantly differ between groups (p = 0.31). In a multivariate model, LAScd and SOFA cv were significantly associated with the occurrence of AF. A LAScd cutoff value of − 11% had a sensitivity of 76% and a specificity of 75% to identify patients with AF. The 30-day cumulative risk of AF was 42 ± 9% with LAScd > − 11% and 8 ± 4% with LAScd ≤ − 11% (log rank *test P* value < 0.0001).

**Conclusion:**

For patients with severe COVID-19 pneumonia, development of AF during ICU stay is common (20%). LAS parameters seem useful in predicting AF within the first 48 h of ICU admission.

*Trial registration*: NCT04354558.

## Background

The outbreak of coronavirus disease 2019 (COVID-19) has caused more than 2 million deaths worldwide. The majority of COVID-19 patients (85%) developed mild illness but up to 15% of them present with severe complications, such as acute respiratory distress syndrome, acute myocardial injury or arrhythmia [1, 2], requiring specific medical treatment in intensive care unit (ICU) [3].

Atrial fibrillation (AF) is frequent in critically ill with an incidence varying from of 1.9 to 43.9% and is associated with a substantial morbidity and mortality [4]. For patients suffering from COVID-19, AF is the most frequently documented arrhythmia [5] with an incidence between 19 and 36% according to the current literature [5, 6] and seems to be higher in non-surviving COVID-19 patients [2, 5, 7].

The involvement of COVID-19 infection in the development of AF is probably due to several complex physiopathological mechanisms and triggers, such as hypoxemia, systemic inflammation, electrolyte abnormalities or alteration of the renin–angiotensin aldosterone system [8, 9]. Moreover, patients with AF and patients with COVID-19 share common risk factors and cardiac comorbidities, such as age, obesity or high blood pressure [3].

Left atrial (LA) strain (LAS) analysis is a non-Doppler echocardiographic method based on LA myocardial deformation that reflects LA contractility [10] and assesses LA function, stiffness and fibrous remodeling [11]. This technique allows to analyze precisely the 3 different phases (reservoir, conduit and contraction) of LA function. The main advantages of LAS compared to Doppler are its angle-independence, the lower reverberations effects, its feasibility and its reproducibility [10]. Bi-dimensional speckle tracking echocardiographic (2D-STE) parameters of LA dysfunction have been associated with AF occurrence in several clinical settings, as ischemic stroke [12] or heart failure [13]. To date, there is no specific report on predictors of AF occurrence in patients with severe COVID-19 pneumonia admitted to ICU. However, given the clinical impact of AF in COVID-19 patients, it seems important to identify echocardiographic parameters that predict AF by detecting early LA myocardial dysfunction.

Our hypothesis is that LA myocardial contractility abnormalities in severe pneumonia related to COVID-19 infection are associated with AF development. This hypothesis was tested using LAS analysis, assessed by transthoracic echocardiography (TTE) and measured by an automated software. The aim of this study was to evaluate the diagnostic ability of LAS parameters to predict occurrence of AF in patients admitted to ICU with severe COVID-19 pneumonia.

## Materials and methods

### Population

Adult patients (> 18 years of age) admitted to ICU at Amiens University Hospital for severe hypoxemic pneumonia related to SARS-Cov2 infection, with a TTE performed in sinus rhythm within 48 h of ICU admission, were prospectively included in the study. Exclusion criteria were patients with permanent AF, permanent atrial and/or ventricular pacing, patients under extracorporeal membrane oxygenation (ECMO), supraventricular tachycardia during the TTE exam and those with poor image quality for LA strain analysis. Patients were included on the day when TTE was performed.

### Ethics

This is an ancillary study of a prospective cohort study of patients with COVID-19 infection hospitalized in ICU at Amiens University Hospital (NCT04354558). This study was approved by the Amiens University Hospital IRB (Comite de Protection des Personnes Nord-Ouest II CHU–Place V. Pauchet, 80054 AMIENS Cedex 1, CNIL Number: PI2020_843_0026). In accordance with French law on clinical research for non-interventional studies, informed consent was waived but oral and written information as provided whenever possible to the patients and systematically to their families specifying that they could oppose the use of their data [14].

### Data

Data from electronical data, medical reports and biological values were collected prospectively. SARS-Cov2 infection was confirmed by a positive Reverse transcription polymerase chain reaction on nasopharyngeal swab or bronchoalveolar lavage on admission to our ICU. The severity of illness at the time of TTE exam was evaluated by the simplified acute physiology score (SAPS) II [15] and the Sequential Organ Failure Assessment (SOFA) score [16]. Vasopressor use was evaluated by the SOFA cardiovascular (SOFA cv) score [16]. Severity of COVID-19 pneumonia was defined according to the World Health Organization (WHO) case definition [17]. The severe group included patients with respiratory distress syndrome (respiratory rate ≥ 30 bpm) and/or oxygen saturation ≤ 93% at rest and/or ratio of arterial partial pressure of oxygen to fractional concentration of oxygen in inspired air < 300 mm Hg and/or > 50% lesion progression over 24–48 h by pulmonary imaging. The critical group included patients with respiratory failure requiring mechanical ventilation and/or with shock or organ failure [17]. The vital status at Day 30 was collected.

### Occurrence of AF

Occurrence of AF was defined by an AF episode lasting at least 30 s recorded by a 12-lead ECG or a single-lead ECG tracing [18] during ICU stay in patients with no prior history of persistent or permanent AF [4]. Patients with history of paroxysmal AF before ICU admission and/or with AF occurrence between ICU admission and time of TTE were not excluded.

All patients were monitored 24-h a day for all hemodynamic parameters including heart rate with 5-lead ECG. Twelve-lead ECG or offline electronic single-lead ECG tracing recorded were analyzed by a cardiac electrophysiologist blinded to the LAS analysis. The risk of ischemic stroke in patients with AF was assessed by the CHA_2_DS_2_-VASc score [18]. The primary endpoint was the occurrence of AF during ICU stay.

### Echocardiography and LA strain analysis

TTE was performed by trained operators in supine position, within 48 h of ICU admission. Standard echocardiography protocol were used in accordance with the American Society of Echocardiography guidelines [19] and the European Society of Cardiology [20]. Echocardiographic images were obtained by a high-quality commercially available ultrasound system (CX 50, Philips Healthcare). All operators had a level III competence of general adult TTE [21].

### LA strain analysis

**Fig. 1:**
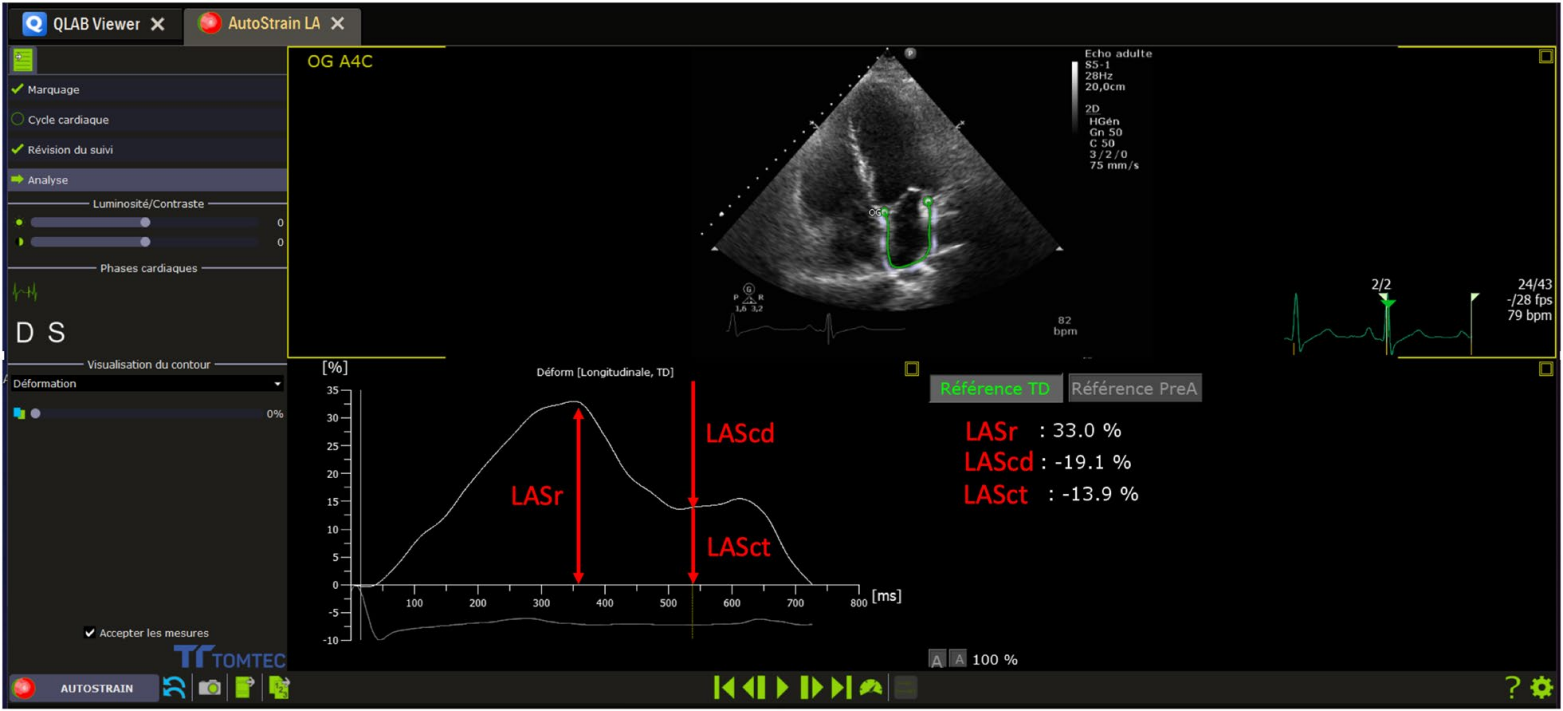
Measurement of LAS parameters with an automated software. LA strain values were automatically measured during the different LA phases: LASr measured as the first peak positive deflection, LAScd measured as the difference between LASr and LASct values, LASct measured as the beginning of the P wave contraction. LAS: left atrial strain. LASr: left atrial strain reservoir phase. LAScd: left atrial strain conduit phase. LASct: left atrial strain contraction phase

LAS analysis was obtained using an automated speckle tracking software (Auto-Strain QLAB 13.0, Philips Medical systems, Andover, MA, USA) with a LAS dedicated mode. The LAS was defined as the strain value in three phases: reservoir strain in systole (LASr), conduit strain in early diastole (LASct) and contraction strain in late diastole (LAScd) [10]. LASr was a positive value, while LASct and LACcd were negative values. LAS values, for each phase, were obtained from an optimized apical four-chamber view using an automated approach as recommended [10] (Fig. 1). The regions of interests (ROI) were generated automatically and LA endocardial border was manually adjusted when required. The QRS complex was used as initial zero-baseline strain electrocardiogram reference point as recommended [10]. All LAS measurements were performed by an experienced cardiologist blind to clinical data.

### LA cycle

LAS values were automatically measured by the software from the LA longitudinal strain curve (Fig. 1). When using the QRS complex as a zero-reference point, the first peak positive deflection corresponds to the value of LA reservoir function. The value of LA contraction function was obtained at the beginning of the P wave contraction. The value of LAS conduit function was calculated as the difference between LASr and LASct values. LA phases definition and LAS measurement were performed according to the European Association of Cardiovascular Imaging (EACVI)/American society of Echocardiography (ASE) guidelines [10].

### Statistical analysis

Data are expressed as mean ± standard deviation (SD), median [interquartile range] or numbers (percentage), as appropriate. Variables were compared between groups (AF and non-AF group) using Mann–Whitney or Chi-square tests, as appropriate. A receiver-operating characteristic curve (ROC) was built to assess the diagnostic performance of LASr, LASct, LAScd for prediction of AF occurrence. Area under ROC curves (AUC) of echocardiographic parameters were compared using Delong’s test. The Youden index was used to determine the optimal threshold of LAS parameters for the prediction of AF occurrence.

To evaluate independent factor associated with AF, univariate and multivariate logistic regression were performed. All factors with a *P* value < 0.10 in univariate analysis were included in the multivariate model. The calibration of the model was assessed by the Hosmer–Lemeshow goodness-of-fit statistic (good fit was defined as a *p* value of > 0.05) [22]. The C-statistic test was used to test the ability of the model to discriminate patients with and without AF [23]. Data are presented as odds ratio (ORs) and 95% confidence intervals (CIs). Cumulative risk curves, as function of time, were generated using the Kaplan–Meier method, and compared by the log-rank test. A statistical test was significant when P value was under 0.05. All *P* values are the results of 2-tailed tests. Statistical analyses were performed using SPSS software version 24 (IBM Corp, Armonk, NY).

### Reproducibility analysis

To evaluate the intra-observer variability for offline LAS analysis, data of 10 patients were randomly selected and analyzed by two operators with at least a 1-week interval between the two analyses. Inter‐observer and intra-observer reproducibility of LAS measurements was assessed using intraclass correlation coefficient (ICC).

## Results

### *Participant’s flow chart (Fig. **2**)*

**Fig. 2:**
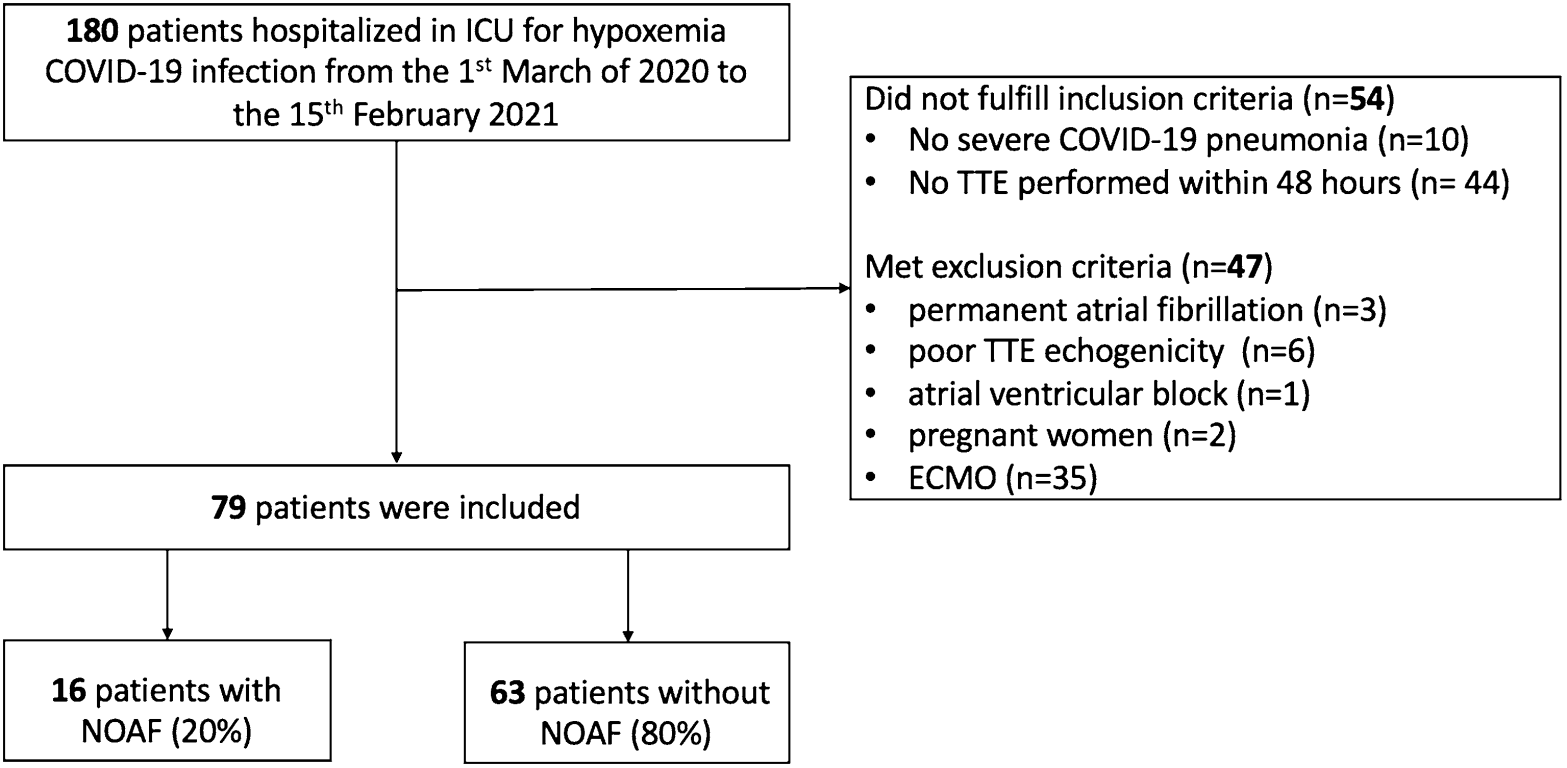
Flow diagram of the study group. AF: new onset atrial fibrillation; ICU: intensive care unit; TTE: transthoracic echocardiography

Between March 1st 2020 and February 15th 2021, 180 patients were admitted in our ICU for COVID-19 infection, 126 patients fulfilled the inclusion criteria and 47 patients were excluded. Especially, 6 patients (13%) were excluded for poor TTE image quality that did not allow LAS analysis. A total of 79 patients were included in the study. The study population was divided in 2 groups according to the presence of AF (AF group and no AF group) during ICU stay.

### Baseline participant characteristics, AF prevalence and outcome (*Table **1* and *Table **2*)

**Table 1:** General characteristic data of the study population

Variables	No AF (*n* = 63)	AF (*n* = 16)	P value
Age (years)	65 [59–70]	73 [65–76]	**0.026**
BMI (kg.m^−2^)	31.5 [24.9–40.7]	29.3 [26.2–32.8]	0.8
SAPS II score during TTE	32 [21–49]	58 [43–62]	**0.001**
Male gender *(n; %)*	40 (65)	14 (88)	0.12
Medical history			
No history	8 (13)	3 (19)	0.68
Hypertension	31 (50)	12 (75)	0.09
Diabetes	13 (21)	6 (38)	0.19
Dyslipidemia	19 (31)	4 (25)	0.76
Smoking (former or active)	20 (33)	4 (25)	0.76
Chronic renal disease	5 (8)	2 (12)	0.62
COPD/asthma	11 (18)	2 (13)	1
Coronary or peripheral artery disease	15 (24)	5 (32)	0.53
Obstructive sleep apnea	5 (8)	1 (6)	1
Chronic treatment			
Statine	19 (31)	7 (44)	0.38
Beta blocker	17 (28)	6 (38)	0.54
ACE inhibitor	11 (18)	5 (32)	0.30
ARBs	14 (23)	3 (18)	1
Diuretic	9 (15)	4 (25)	0.45
Aspirin	14 (23)	6 (38)	0.33
Metformin	9 (15)	4 (25)	0.45
Time to first symptom to ICU admission (days)	8 [5–11]	8 [4–14]	0.56
COVID 19 specific treatment (n = 67/89)			
Dexamethasone	49 (81)	11 (74)	0.70
Remdesevir	3 (5)	0	1
Tocillizumab	1 (2)	0	1
Atrial fibrillation			
Previous paroxysmal AF	6 (10)	2 (12)	0.61
Occurrence of AF, (days)	–	7 [2–11]	–
CHA_2_DS_2_-VASc	2 [1–3]	3 [1–4]	0.20
Outcome			
Arterial thromboembolic events	0	1 (6)*	0.21
Length under MV, *days*	17 [10–24]	28 [16–44]	** < 0.001**
Mortality at 30-days	14 (22)	7 (43)	0.11
ICU discharge, *days*	61 (93)	13 (82)	0.06
Length of stay in ICU, *days*	12 [5–21]	25 [14–33]	** < 0.001**

Bold indicates *P* < 0.05

Continuous variables are expressed as median [interquartile range] and categorical variables as number (percentage)

*ACE* angiotensin-converting enzyme, *AF* atrial fibrillation, *ARBs* angiotensin II receptor blockers, *BMI* body mass index, *COPD* chronic obstructive pulmonary disease, *ICU* intensive care unit, *MV* mechanical ventilation, *SAP* systolic arterial pressure, *SAPS* simplified acute physiology score, *TTE* transthoracic echocardiography

^*^ischemic stroke

**Table 2:** Hemodynamics parameters and biological investigations during TTE exam

Hemodynamic			
HR, *bpm*	82 [71–90]	78 [59–103]	0.66
SAP, *mmHg*	128 [115–138]	120 [110–131]	0.11
DAP, *mmHg*	65 [59–73]	62 [53–71]	0.27
MAP, *mmHg*	86 [76–94]	85 [71–91]	0.46
SpO_2_, *%*	93 [91–96]	93 [91–96]	0.74
T, *°Celcius*	37.6 [36.7–38.3]	36.8 [36.4–37.7]	0.07
SOFA score during TTE	3 [2–5]	5 [3–10]	0.002
SOFA cardiovascular score during TTE	0 [0–1]	4 [1–4]	0.0001
Critical group (n = 25)			
Norepinephrine use, *n (%)*	14 (22)	11 (69)	**< 0.001**
* Norepinephrine (ug/Kg/min)*	0.26 [0.12–0.96]	0.15 [0.12–0.28]	0.56
Mechanical ventilation, *n (%)*	25 (41)	13 (81)	**0.005**
* PEEP (cmH*_*2*_ *O)*	10 [7–12]	11 [9–14]	0.73
PaO_2_, *mmHg*	79 [65–98]	87 [68–120]	0.96
Biological investigations			
WBC, *mm*^*−3*^	9000 [6100–12900]	7300 [5850–13525]	0.47
Lymphocyte count, *mm*^*−3*^	700 [400–1250]	800 [300–1100]	0.84
Hemoglobin, *g l*^*−1*^	12.6 [11.1–13.4]	12.0 [11.4–13.6]	0.90
C reactive protein, *mg l*^*−1*^	132 [97–220]	152 [144–195]	0.52
Creatinin, umol l^−1^	71 [58–90]	82 [56–154]	0.23
Troponine Tc HS, *ng ml*^*−1*^	18 [6–42]	48.5 [3.25–148]	0.48
BNP, *pg ml*^*−1*^	66 [42–123]	119 [40–185]	0.42

Bold indicates *P* < 0.05

Continuous variables are expressed as median [interquartile range]

*BNP* brain natriuretic peptide, *COPD* chronic obstructive pulmonary disease, *DAP* diastolic arterial pressure, *HR* heart rate, *MAP* mean arterial pressure, *PEEP* positive end-expiratory pressure, *SAP* systolic arterial pressure, *SOFA* sequential organ failure assessment, *Sp0*_*2*_ pulse saturation of oxygen, *TTE* transthoracic echocardiography, *WBC* white blood cell

Medical history, chronic treatment, time to ICU admission (from first symptoms), biological investigations and hemodynamic parameters were comparable between the two groups. AF was documented in 16/79 patients (20%) with a median time of 7 [2–11] days (Table 1**)** from ICU admission. In the AF group, patients were older (73 [65–76] vs. 65 [59–70] years; *P* = 0.026) than in the no AF group. SAPS II score at ICU admission was higher for the AF group (58 [43–62] vs. 32 [21–49], *P* < 0.0001) than for the no AF group. In the AF group, there was significantly more critical patients (n = 11/16[69%] vs. n = 14/63[22%], *P* < 0.001) according to the WHO definition. Moreover, patients of the AF group had higher SOFA cv score than patients of the no AF group (4 [1–4] vs. 0 [0–1]*, P* = 0.0001).

In the AF group, duration of mechanical ventilation was longer (28 [16–44] vs. 17 [10–24] days, *P* < 0.0001) as well as ICU length of stay (25 [14–33] vs. 12 [5–21] days, *P* < 0.001) compared to no AF group. However, there was no difference in mortality at 30 days between the two groups (n = 7/16 [43%] vs. 14/63 [22%], *P* = 0.11, respectively, for AF and no AF group).

### LAS parameters and AF (*Table **3*)

**Table 3:** Echocardiographic data

Overall population (*n* = 79)	No AF (*n* = 63)	AF (*n* = 16)	*P* value
LV systolic parameters			
LVEF (%)	61 [51–69]	66 [53–70]	0.37
LV end diastolic volume (ml)	109 [74–129]	109 [90–156]	0.50
LV end systolic volume (ml)	43 [26–58]	38 [24–65]	0.85
Stroke volume index (ml/m^2^)	33 [26–39]	27 [24–35]	0.12
Cardiac index (l/min/m^2^)	2.49 [1.98–3.23]	2.15 [1.78–3.01]	0.24
LV diastolic functional parameters			
E wave (cm s^−1^)	84 [69–92]	77 [65–98]	0.60
A wave (cm s^−1^)	83 [68–105]	74 [61–100]	0.63
E/A ratio	0.9 [0.74–1.2]	0.8 [0.7–1.2]	0.74
Lateral E/e’	8.5 [6.3–10.4]	9.7 [7.4–12.0]	0.24
E wave deceleration time (ms)	250 [180–309]	254[190–304]	0.93
LA volume (ml)	49 [39–58]	56 [32–67]	0.52
LA volume index (ml/m^2^)	23 [18–27]	28 [16–38]	0.19
RV Parameters			
RV basal dimension (mm)	46 [40–51]	43 [41–50]	0.72
RV mid-cavity dimension (mm)	34 [29.40]	32 [26–39]	0.38
RV longitudinal dimension (mm)	77 [71–82]	74 [71–78]	0.42
RV EDA (cm^2^)	20 [15–25]	18 [16–21]	0.37
RV ESA (cm^2^)	11 [7–15]	9 [8–11]	0.18
RA volume indexed to BSA (ml/m^2^)	21 [14–25]	19 [15–25]	0.95
RV systolic function parameters			
TAPSE (mm)	24.2 [21.0–28]	21.5 [18.2–24.1]	0.6
RV- S’ (cm/s-1)	16.0 [13.4–19.4]	18.0 [16.3–20.0]	0.43
RV FAC (%)	47 [38–53]	49 [46–53]	0.23
Pericardial effusion (> 10 mm)	3 (5)	2 (12)	0.26
Valvular heart disease	2 (3)†	1 (6)*	0.10
LA strain parameters			
LASr (%)	30.5 [23.8–36.2]	20.2 [12.3–27.3]	**0.002**
LAScd (%)	− 17.2 [(− 5.0)–(− 10.2)]	− 8.1 [(− 6.3)–(− 10.9)]	** < 0.001**
LASct (%)	− 13.3 [(− 7.7)–(− 16.9)]	− 9.7 [(− 5.2)–(− 16.1)]	0.31

Bold indicates *P* < 0.05

*CO* cardiac output, *EDA* end diastolic area, *ESA* end systolic area, *FAC* fractional area change, *LA* left atrial, *LAScd* left atrial strain during conduit phase, *LASct* left atrial strain during contraction phase, *LASr* left atrial strain during reservoir phase, *LV* left ventricle, *LVEF* left ventricular ejection fraction, *RA* right atrium, *RV* right ventricle, *TAPSE* tricuspid annular plane systolic excursion

*one moderate mitral regurgitation by prolapse

†one severe mitral regurgitation and one moderate aortic regurgitation

Regarding echocardiographic parameters, only LAS parameters were significantly different between the 2 groups. In the AF group, LASr and LAScd were significantly impaired compared to the other group (− 20.2 [− 12.3; − 27.3] vs. -30.5 [− 23.8; − 36.2] %, *P* = 0.002 and − 8.1 [− 6.3; − 10.9] vs. − 17.2 [− 5.0; − 10.2] %, *P* < 0.0001, respectively)). LASct did not significantly differ between groups (*P* = 0.31).

## LAScd as a predictor of AF (Fig. 3)

**Fig. 3:**
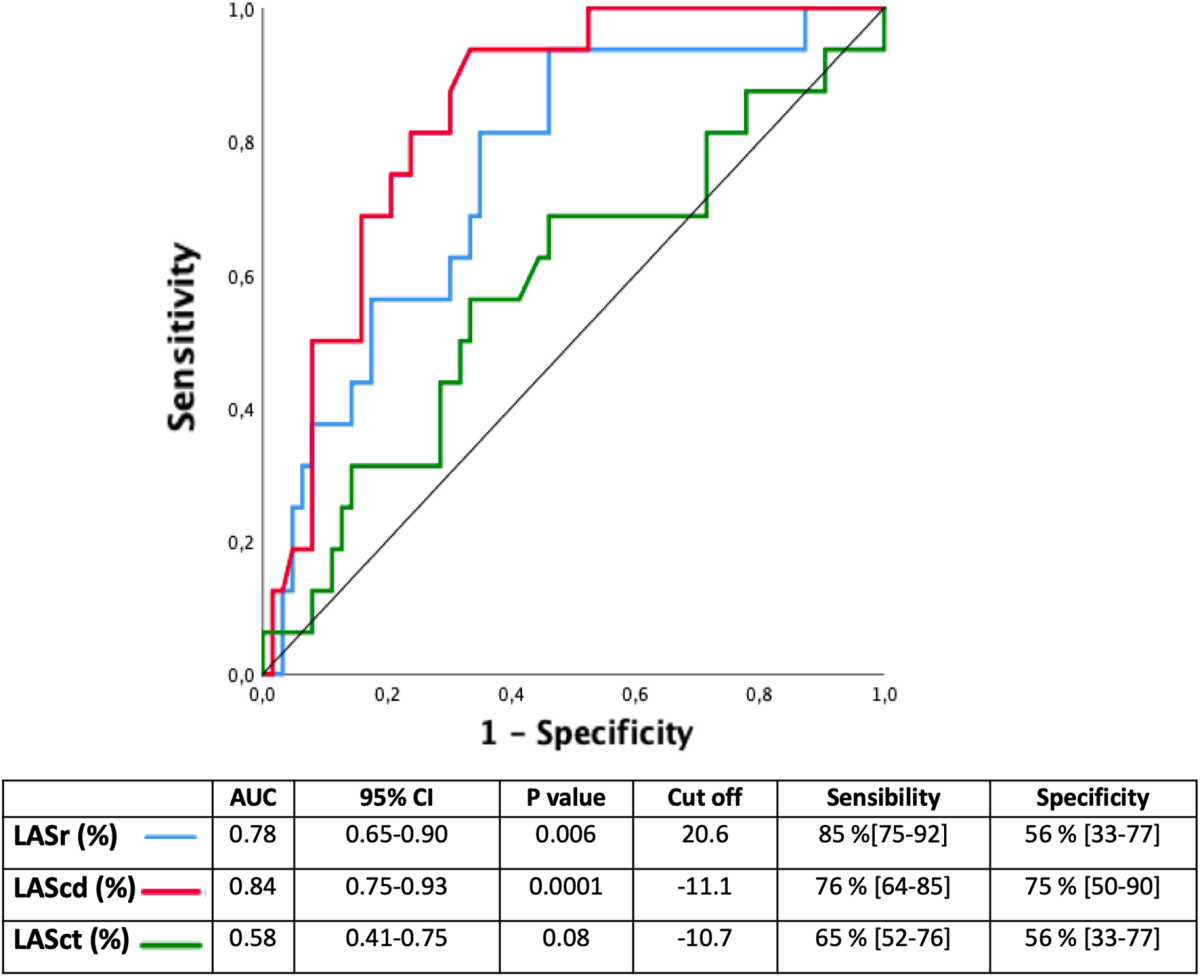
ROC curve analysis of LAS parameters for predicting AF. AF: atrial fibrillation; LAS: left atrial strain

The comparison of ROC curves showed that LAScd had the highest AUC to predict AF compared to other LAS parameters**.** A LAScd cutoff value of − 11.1% had a sensitivity of 76% (95% CI [64–85]) and a specificity of 75% (95% CI [50–90], with an AUC of 0.84 [0.75–0.93], *P* < 0.0001, to identify patients with AF.

### Multivariate model analysis (Table 4)

**Table 4:** Factors associated with occurrence of AF in pneumonia related to COVID-19 infection

Variable	Univariate analysis		Multivariate analysis	
	OR (95%CI)	*P*	OR (95%CI)	*P*
LAScd (for each 1% increase)	1.27 [1.10–1.47]	0.001	1.24 [1.04–1.48]	**0.018**
LASr (for each 1% increase)	0.89 [0.83–0.95]	0.002	NS	
SOFA cv > 1	6.4 [1.96–20.9]	0.002	5.56 [1.41–22.11]	**0.015**
PEEP	0.86 [0.68–1.09]	0.86	NR	–

Bold indicates *P* < 0.05

**Fig. 4:**
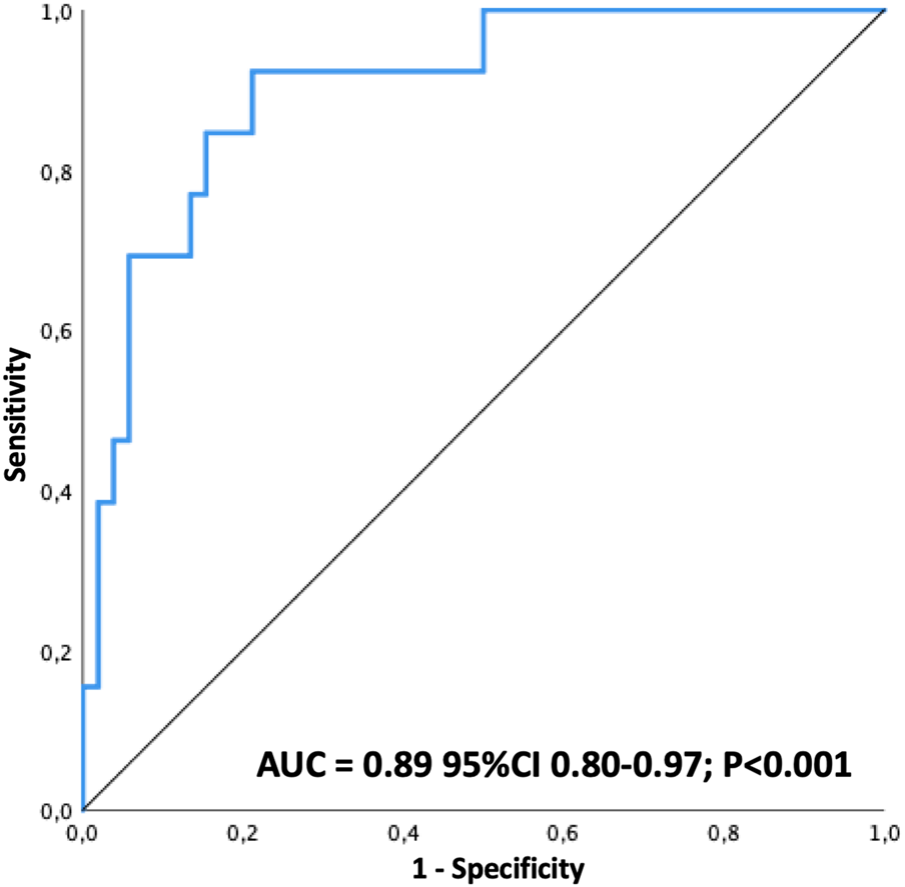
C-statistic and receiver operating characteristics curve (ROC) of factors associated with the occurrence of AF. The ROC curve analyzed the discrimination ability of the model composed of LAScd and SOFA cv > 1 to predict AF. AF: atrial fibrillation; AUC: area under curve; CV: cardiovascular; ROC: receiver operating characteristic curve. SOFA: sequential organ failure assessment

The multivariable model showed a good calibration as assessed by the Hosmer and Lemeshow goodness of fit test [p = 0.98] and a fair discrimination as assessed by the receiver operating characteristics curve [area under the curve (AUC) 0.89; 95% CI 0.80–0.97; p < 0.001] (Fig. 4)

*CI* confidence interval, *CV* cardiovascular, *LAScd* left atrial strain during conduit phase, *SOFA* sequential organ failure assessment, *PEEP* positive end-expiratory pressure. *NR* not retained in the final model, *NS* not significant in the final model

**Fig. 5:**
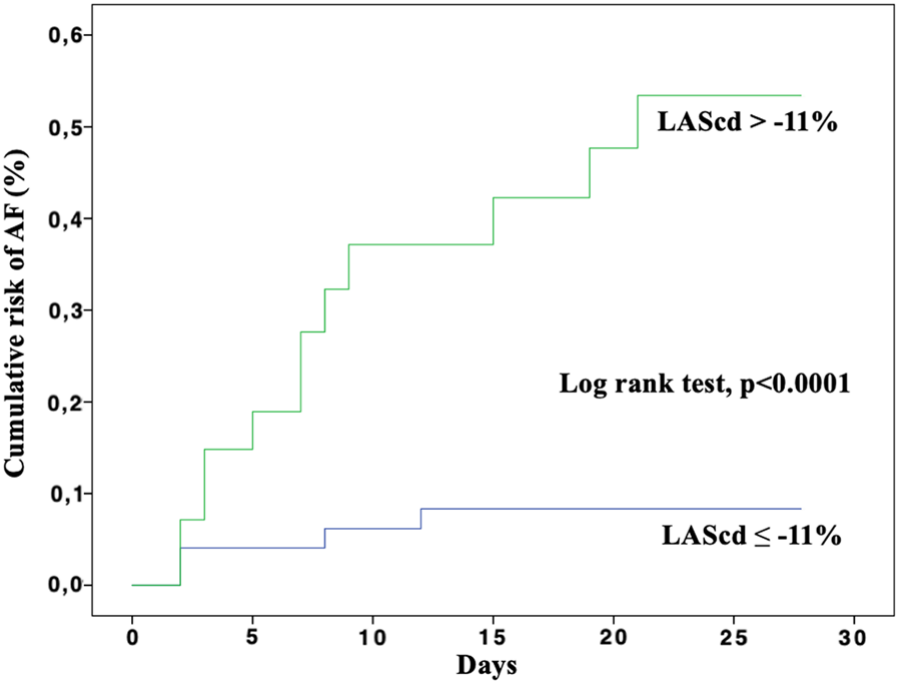
Cumulative risk of AF according to LAScd. AF: atrial fibrillation; LAScd: left atrial strain conduit phase

In a multivariate model (Table 4), only LAScd and SOFA cv > 1 were independently associated to the occurrence of AF with an OR of 1.24 [95% CI 1.04 to 1.48] and 5.56 [95% CI 1.41 to 22.11], respectively. The discrimination ability of the model using C-statistics showed an AUC of 0.89 (95% CI 0.80 to 0.97) (Fig. 4). The 30-day cumulative risk of AF was 42 ± 9% with LAScd > − 11% and 8 ± 4% with LAScd ≤ − 11% (log rank test *P* value < 0.0001) (Fig. 5).

### LA strain analysis reproducibility (Table 5)

**Table 5:** Reproducibility of LA strain analysis with an automated software

LA strain parameters	ICC for inter-operator	95% CI	ICC for intra-operator	95% CI
LASr	0.97	0.84–0.99	0.96	0.84–0.99
LAScd	0.86	0.52–0.96	0.94	0.78–0.98
LASct	0.89	0.74–0.98	0.90	0.61–0.97

*CI* confidence interval, *ICC* intraclass correlation coefficient, *LA* left atrial, *LAScd* left atrial strain during conduit phase, *LASct* left atrial strain during contraction phase, *LASr* left atrial strain during reservoir phase

The reproducibility of LAScd measurement had a very strong correlation with an ICC of 0.86 (95% CI 0.52–0.96) for the inter-operator reproducibility and 0.94 (95% CI 0.74–0.98) for the intra-operator reproducibility.

## Discussion

Our results showed that 20% of COVID-19 patients admitted to ICU developed AF during ICU stay. LAS parameters notably LAScd were impaired in patients with AF. LAScd cutoff value of − 11% seems to be accurate to identify patients with high risk of AF during ICU stay. We also showed that LAS analysis is feasible and highly reproducible in ICU setting using an automated software.

### AF and critical COVID-19 patients

Recent findings supported the higher likelihood of observing AF in COVID-19 patients admitted to the ICU [24]. Moreover, AF may worsen the clinical evolution of pneumonia in these patients [25]. Colon et al*.* reported an AF prevalence of 16.5% in ICU patient and showed that mechanical ventilation was strongly associated with AF [24]. Here, we reported comparable results as 20% of our patients developed AF during ICU stay and AF was strongly associated with a more critical state (69% vs. 22%; *P* < 0.0001). Pletzer et al*.* reported an in-hospital mortality of 39.2% (n = 65/166) in COVID-19 patients with AF and showed that AF was an independent predictor of in-hospital mortality [26]. However, in this report only 60% of patients with AF were hospitalized in ICU [18].

### AF and critical ill

AF is a common complication of critical illness and is an independent predictor of mortality [27]. In septic patients, mechanical ventilation, organ failure and norepinephrine use were strongly associated with AF [4]. In our study, norepinephrine use was strongly associated with AF to the contrary to mechanical ventilation. However, AF was not associated with 30-day mortality (43% vs. 22%; *P* = 0.11) probably due to a lack of statistical power explained by the limited sample size.

### AF and LA strain function

AF is associated with adverse outcomes in COVID-19 patients [5, 17]. Therefore, the prediction of AF is of paramount clinical importance. In our study, LAScd was a strong predictor of AF and the identified cutoff value of − 11% was closed to that of previously observed cutoff in different cardiovascular disease. For example, in Chagas disease, the LA conduit function (− 12.6 ± 5.7%) was reported to be a strong predictor of AF [28] due to the depression of the LA conduit function [29]. In a cohort of ischemic stroke, Rasmussen et al*.*, demonstrated that LAScd was worse for the AF group (− 12 ± 5 vs. − 16 ± 7%, *P* < 0.003) compared to patients without AF [12].

LAS reservoir parameter is also a prognostic factor for the occurrence of AF in ischemic stroke, heart failure or after cardiac surgery [12, 13, 30] and reflects LA compliance [11]. Several studies suggested that impaired LA reservoir function may be a sign of LA remodeling, caused by several cardiovascular conditions, such as hypertension, diabetes or ischemic heart disease [12]. In our study, LASr values were significantly impaired in the AF group 30.5 [23.8–36.2] % vs. 20.2 [12.3–27.3] %; *P* = 0.002). Goerlich et al*.* reported similar LASr values (30.4 [26.1–35.8] % vs. 22.3 [20.6–27.8] %; P < 0.001) and shown that LASr parameter was an independent factor of AF in COVID-19 patients [31]. However, in our study, only LAScd remained independently associated with AF probably due to the limited sample size of our cohort.

### LAScd and left ventricular filling pressure in COVID-19 patients

Clinical data on LA mechanistic dysfunction suggested a strong link between left ventricular diastolic dysfunction and risk of AF [32, 33]. LAS analysis, especially LAScd, has recently emerged as a powerful tool for left ventricular diastolic dysfunction evaluation [34] especially when left ventricular end diastolic pressure (LVEDP) was increased [35]. Severe hypoxemic COVID-19 pneumonia may be associated with diastolic dysfunction and/or increased LVEDP. Indeed, COVID-19 infection can lead to myocardial diastolic dysfunction [36] by direct virus related-myocardial injury, inflammation or cardiac fibrosis [37]. COVID-19 may unmask subclinical LA dysfunction or exacerbate preexisting LA dysfunction [38]. Moreover, recent findings suggested that COVID-19 patients with severe respiratory failure had a high prevalence of increased LVEDP [39]. All these elements may lead to AF. However, data about the potential effect of COVID-19 on LAcd function are currently lacking and further studies on the subject would be of great interest.

### Feasibility of LAS analysis in ICU

In this study, we found a high feasibility of LAS parameters in patients with respiratory failure as only 6 patients were excluded for poor image quality. Data on LAS analysis in ICU are scarce. Hence, the present study emphasizes the fact that LAS analysis can be easily performed in ICU patients using a dedicated mode for LAS analysis and an automated approach as recommended [10].

## Limitations

The first limitation of our study is the limited sample size especially in the AF group. Second, LAS analysis was calculated only from a single four-chamber cardiac view. Computation of the biplane LAS (four-chamber and two-chamber view) would have provided more data on LA function. However, the use of a single apical view is currently recommended for a greater feasibility [10]. Third, we were faced with the known limitations factors of LAS analysis (far field, pulmonary veins and LA appendage orifice, LA thin walls) [40] leading to the exclusion of 6 patients from the study.

In our study, LAS measurement was strongly reproducible probably due to the fact that LAS analysis was performed by an echocardiography expert (level III competence according to the EACVI definition [21]). LAS performed by an operator with a lower skill level could alter the validation of our results in further studies. To have clinical consistency on COVID-19 infection and early virus-related myocardial injury, only patients who had a TTE within 48 h of ICU admission were included. The exclusion of these patients might have led to a selection bias. However, a TTE performed after this delay would be difficult to interpret, especially because of the potential fluid overload related to initial resuscitation.

In our study, norepinephrine use was associated with AF. Norepinephrine is known to increases cardiac preload [41] which can probably impair the LAS value. Indeed, as left ventricular strain, LAS is probably influenced by loading conditions and abnormal LAS values are not necessarily synonymous of LA dysfunction [42]. Loading conditions and compensatory LA remodeling influence each other. It is, therefore, difficult to determine the contractile state of the LA myocardium from a single strain measurement. However, LAS analysis remains very sensitive to identify LA functional changes in clinical practice [10].

Finally, the sensitivity and specificity values for LAScd were derived from our population study and require external validation. However, we used a standard method [10] and full automated analysis of LAS which allowed a good reproducibility and reduced the risk of error.

## Conclusion

In patients with severe COVID-19 pneumonia, development of AF during the ICU stay is common (20%). LAS parameters seem useful in predicting AF within the first 48 h of ICU admission. Further studies with larger sample size investigating the relationship between AF and LAS parameters in COVID-19 patients are required.

## Data Availability

The data sets used and/or analyzed during the current study are available from the corresponding author on reasonable request.

## Abbreviations

2D-STE: Bi-dimensional speckle tracking echocardiography
AF: Atrial fibrillation
COVID-19: Coronavirus infectious disease 2019
ICU: Intensive care unit
LA: Left atrial
LAS: Left atrial strain
LASr: Left atrial strain during the reservoir phase
LAScd: Left atrial strain during the conduit phase
LASct: Left atrial strain during the contractile phase
LVEDP: Left ventricular end diastolic pressure

## References

[1] Guo T, Fan Y, Chen M (2020). Cardiovascular implications of fatal outcomes of patients with coronavirus disease 2019 (COVID-19). JAMA Cardiol.

[2] Wang D, Hu B, Hu C (2020). Clinical characteristics of 138 hospitalized patients with 2019 novel coronavirus-infected pneumonia in Wuhan, China. JAMA.

[3] COVID-ICU Group on behalf of the REVA Network and the COVID-ICU Investigators (2021). Clinical characteristics and day-90 outcomes of 4244 critically ill adults with COVID-19: a prospective cohort study. Intensive Care Med.

[4] Wetterslev M, Haase N, Hassager C (2019). New-onset atrial fibrillation in adult critically ill patients: a scoping review. Intensive Care Med.

[5] Inciardi RM, Adamo M, Lupi L (2020). Characteristics and outcomes of patients hospitalized for COVID-19 and cardiac disease in Northern Italy. Eur Heart J.

[6] Gawałko M, Kapłon-Cieślicka A, Hohl M (2020). COVID-19 associated atrial fibrillation: Incidence, putative mechanisms and potential clinical implications. Int J Cardiol Heart Vasc.

[7] Pardo Sanz A, Salido Tahoces L, Ortega Pérez R (2021). New-onset atrial fibrillation during COVID-19 infection predicts poor prognosis. Cardiol J.

[8] Hu Y-F, Cheng W-H, Hung Y (2020). Management of atrial fibrillation in COVID-19 pandemic. Circ J.

[9] Russo V, Rago A, Carbone A (2020). Atrial fibrillation in COVID-19: from epidemiological association to pharmacological implications. J Cardiovasc Pharmacol.

[10] Badano LP, Kolias TJ, Muraru D (2018). Standardization of left atrial, right ventricular, and right atrial deformation imaging using two-dimensional speckle tracking echocardiography: a consensus document of the EACVI/ASE/Industry Task Force to standardize deformation imaging. Eur Heart J Cardiovasc Imaging.

[11] Cameli M, Mandoli GE, Loiacono F (2016). Left atrial strain: a useful index in atrial fibrillation. Int J Cardiol.

[12] Rasmussen SMA, Olsen FJ, Jørgensen PG (2019). Utility of left atrial strain for predicting atrial fibrillation following ischemic stroke. Int J Cardiovasc Imaging.

[13] Park JJ, Park J-H, Hwang I-C (2020). Left atrial strain as a predictor of new-onset atrial fibrillation in patients with heart failure. JACC Cardiovasc Imaging.

[14] Toulouse E, Masseguin C, Lafont B (2018). French legal approach to clinical research. Anaesth Crit Care Pain Med.

[15] Le Gall JR (1993). A new simplified acute physiology score (SAPS II) based on a European/North American multicenter study. JAMA.

[16] Vincent JL, Moreno R, Takala J (1996). The SOFA (Sepsis-related Organ Failure Assessment) score to describe organ dysfunction/failure. On behalf of the Working Group on Sepsis-Related Problems of the European Society of Intensive Care Medicine. Intensive Care Med.

[17] Wu Z, McGoogan JM (2020). Characteristics of and Important Lessons From the Coronavirus Disease 2019 (COVID-19) Outbreak in China: Summary of a Report of 72 314 Cases From the Chinese Center for Disease Control and Prevention. JAMA.

[18] Hindricks G, Potpara T, Dagres N (2021). 2020 ESC Guidelines for the diagnosis and management of atrial fibrillation developed in collaboration with the European Association for Cardio-Thoracic Surgery (EACTS). Eur Heart J.

[19] Lang RM, Badano LP, Mor-Avi V (2015). Recommendations for cardiac chamber quantification by echocardiography in adults: an Update from the American Society of Echocardiography and the European Association of Cardiovascular Imaging. J Am Soc Echocardiogr.

[20] Adler Y, Charron P, Imazio M (2015). 2015 ESC Guidelines for the diagnosis and management of pericardial diseases: The Task Force for the Diagnosis and Management of Pericardial Diseases of the European Society of Cardiology (ESC) Endorsed by: The European Association for Cardio-Thoracic Surgery (EACTS). Eur Heart J.

[21] Popescu BA, Andrade MJ, Badano LP (2009). European Association of echocardiography recommendations for training, competence, and quality improvement in echocardiography. Eur J Echocardiogr.

[22] Lemeshow S, Hosmer DW (1982). A review of goodness of fit statistics for use in the development of logistic regression models. Am J Epidemiol.

[23] Romero C, te Velde L, Ponsen H, Cleophas TJ (2012). C-statistics versus logistic regression for assessing the performance of qualitative diagnostic tests. Clin Chem Lab Med.

[24] Colon CM, Barrios JG, Chiles JW (2020). Atrial arrhythmias in COVID-19 patients. JACC Clin Electrophysiol.

[25] Bhatla A, Mayer MM, Adusumalli S (2020). COVID-19 and cardiac arrhythmias. Heart Rhythm.

[26] Peltzer B, Manocha KK, Ying X (2020). Outcomes and mortality associated with atrial arrhythmias among patients hospitalized with COVID-19. J Cardiovasc Electrophysiol.

[27] Kanjanahattakij N, Rattanawong P, Krishnamoorthy P (2019). New-onset atrial fibrillation is associated with increased mortality in critically ill patients: a systematic review and meta-analysis. Acta Cardiol.

[28] Saraiva RM, Pacheco NP, Pereira TOJS (2020). Left atrial structure and function predictors of new-onset atrial fibrillation in patients with chagas disease. J Am Soc Echocardiogr.

[29] Saraiva RM, Demirkol S, Buakhamsri A (2010). Left atrial strain measured by two-dimensional speckle tracking represents a new tool to evaluate left atrial function. J Am Soc Echocardiogr.

[30] Pessoa-Amorim G, Mancio J, Vouga L (2018). Impaired left atrial strain as a predictor of new-onset atrial fibrillation after aortic valve replacement independently of left atrial size. Rev Esp Cardiol.

[31] Goerlich E, Minhas A, Gilotra N (2021). Left atrial function in patients with COVID-19 and its association with incident atrial fibrillation/flutter. J Am Soc Echocardiogr.

[32] Rosenberg MA, Manning WJ (2012). Diastolic dysfunction and risk of atrial fibrillation: a mechanistic appraisal. Circulation.

[33] Labbé V, Ederhy S, Lapidus N (2021). Transesophageal echocardiography for cardiovascular risk estimation in patients with sepsis and new-onset atrial fibrillation: a multicenter prospective pilot study. Ann Intensive Care.

[34] Fan J-L, Su B, Zhao X (2020). Correlation of left atrial strain with left ventricular end-diastolic pressure in patients with normal left ventricular ejection fraction. Int J Cardiovasc Imaging.

[35] von Roeder M, Rommel K-P, Kowallick JT (2017). Influence of left atrial function on exercise capacity and left ventricular function in patients with heart failure and preserved ejection fraction. Circ Cardiovasc Imaging.

[36] Freaney PM, Shah SJ, Khan SS (2020). COVID-19 and heart failure with preserved ejection fraction. JAMA.

[37] Helms J, Combes A, Aissaoui N (2021). Cardiac injury in COVID-19. Intensive Care Med.

[38] Song L, Zhao S, Wang L (2020). Cardiovascular changes in patients with COVID-19 from Wuhan, China. Front Cardiovasc Med.

[39] Caravita S, Baratto C, Di Marco F (2020). Haemodynamic characteristics of COVID-19 patients with acute respiratory distress syndrome requiring mechanical ventilation. An invasive assessment using right heart catheterization. Eur J Heart Fail.

[40] Voigt J-U, Mălăescu G-G, Haugaa K, Badano L (2020). How to do LA strain. Eur Heart J Cardiovasc Imaging.

[41] Monnet X, Jabot J, Maizel J (2011). Norepinephrine increases cardiac preload and reduces preload dependency assessed by passive leg raising in septic shock patients. Crit Care Med.

[42] Voigt J-U, Cvijic M (2019). 2- and 3-Dimensional myocardial strain in cardiac health and disease. JACC Cardiovasc Imaging.

